# Topological Complexity of the Length-Constrained Systems of Finite Symbols

**DOI:** 10.3390/e28070801

**Published:** 2026-07-14

**Authors:** Qingsong Wang, Cailing Yao, Jiaxing He, Bingzhe Hou

**Affiliations:** School of Mathematics, Jilin University, Changchun 130012, China; qswang21@mails.jlu.edu.cn (Q.W.); 13578632725@163.com (C.Y.); susanhjx@163.com (J.H.)

**Keywords:** constrained systems, topological entropy, subshifts of finite type, transition matrix, 37B10, 37B40, 94A55, 94A17

## Abstract

In this paper, we consider a class of constrained systems named *n*-tuple upper bound $\left(m_{1},\cdots ,m_{n}\right)$-constrained systems (*n*-TUB systems briefly) for 2≤n<∞, which are subshifts of finite type. We determinate the topological entropies (Shannon capacities) $C(m_{1},\cdots ,m_{n})$ of all *n*-TUB systems and consequently order all *n*-TUB systems according to the size of the topological entropies. An algorithm is also presented to compute the transition matrix and topological entropy for *n*-TUB systems.

## 1. Introduction

Driven by the needs of the data storage industry, innovations in practical code design have evolved alongside a rigorous mathematical framework for constrained systems. Among these, run-length-limited (RLL) constraints play a key role and are widely employed in magnetic and optical recording systems.

Given any two non-negative integers *d* and *k* with d<k, a binary sequence is said to be (d,k)-constrained if every run of zeros has length at most *k* and any two successive ones are separated by a run of zeros of length at least *d*. A (d,k)-constrained system (also called (d,k)-constrained code) is defined to be the set of all finite-length (d,k)-constrained binary sequences. The above definition can be extended to the case k=∞ by not imposing an upper bound on the lengths of zero-runs.

The history of constrained coding dates back to 1948, when Shannon [1] represented a constrained sequence via a finite state transition diagram (FSTD) and derived the capacity under a constraint. run-length-limited (RLL) codes were introduced by Tang and Bahl [2] in 1970 to support the evolution of magnetic recording at that time. As it is well known, the Shannon capacity plays a major role in the research of (d,k)-RLL systems [3]; in fact, the Shannon capacity is the topological entropy of shift on a (d,k)-RLL system. Shannon capacity and topological entropy are usually used to characterize the topological complexity of systems. Following from [1], the Shannon capacity *C* of a discrete channel is given by

$$C=\lim_{T\to \infty }\frac{\log N(T)}{T},$$

where N(T) is the number of allowed signals of duration *T*. In the language of symbolic dynamical systems, N(T) is just the number of allowed *T*-length codes. R. Adler, A. Konheim and M. McAndrew [4] introduced the concept of topological entropy via open covers in 1965. Later, R. Bowen [5] gave the definition of metric entropy, which coincides with the definition of topological entropy on compact metric spaces. Furthermore, the definition of entropy in symbolic dynamical systems is mathematically identical to that of Shannon entropy (see Definition 4.1.1 in [6]).

Constrained systems, or constrained codes, are widely applied and have also advanced considerably in theory. They have been extensively employed in one-dimensional (1D) magnetic recording devices, including both early peak-detection-based devices and modern sequence-detection-based devices [7,8]. S. Zheng, Y. Liu, and P. Siegel in [9] also found that the constrained codes can be combined with robust signal detection through machine learning frameworks. Driven by the increasing demand for high-density storage in smaller size and with recent developments in page-oriented storage systems, multidimensional constraints are also concerned. Numerous studies have focused on magnetic recording (e.g., [10,11]). Recently, some authors have considered the theory of RLL systems such as the convergence to the capacity as well as the lower and upper bounds on the capacity [12,13,14].

Y. Ma and B. Hou [15] considered a special kind of constrained systems named “double upper bound (p,q)-constrained systems”, which are similar to but different from run-length-limited constrained systems. They determinated the topological entropy (Shannon capacity) C(p,q) of all (p,q)-DUB systems and consequently ordered all (p,q)-DUB systems according to the size of topological entropy. The (∞,∞)-DUB system is the full two-shift, a bilateral (p,∞)-DUB system is a (0,p)-RLL system for every positive integer *p*, a bilateral (1,q)-DUB system is a (1,q)-RLL system for every positive integer *q*, and other (p,q)-DUB systems are not RLL systems.

In this article, we consider a more general case, that is, the “n-tuple upper bound $\left(m_{1},\cdots ,m_{n}\right)$-constrained systems” for n≥2, or briefly *n*-TUB systems. An *n*-TUB system is the set of all n-tuple upper bound (p,q)-constrained bilateral or unilateral sequences and the shift on it. Given *n* positive integers (may be ∞) $m_{1},\cdots ,m_{n}$, we say that a bilateral or unilateral {1,⋯,n}-sequence is *n*-tuple upper bound $\left(m_{1},\cdots ,m_{n}\right)$-constrained if the run length of *k* is no more than $m_{k}$ for k=1,⋯,n. In particular, the (∞,⋯,∞)-TUB system of *n* symbols is the full *n*-shift.

Let $S(m_{1},\cdots ,m_{n})$ be a bilateral or unilateral *n*-TUB system, $1\leq m_{1}\leq \cdots \leq m_{n}\leq \infty$. Denote by $C(m_{1},\cdots ,m_{n})$ the topological entropy or Shannon capacity of $S(m_{1},\cdots ,m_{n})$. Let $A_{p}$ be the number of *p*-length codes in $S(m_{1},\cdots ,m_{n})$. Then,

$$C\left(m_{1},\cdots ,m_{n}\right)=\lim_{p\to \infty }\frac{1}{p}\ln A_{p}.$$

Our main results are as follows.

**Main Theorem**

1. For *n*-TUB system S(∞,∞⋯,∞) and (n+1)-TUB system S(1,1⋯,1,1), we have
  C(∞,∞⋯,∞)=C(1,1⋯,1,1)=lnn.
2. For $n_{1}$-TUB system $S(m_{1},\cdots ,m_{n_{1}})$ and $n_{2}$-TUB system $S(m_{1}^{\prime },\cdots ,m_{n_{2}}^{\prime })$, if $n_{1}<n_{2}$, then
  $$C\left(m_{1},\cdots ,m_{n_{1}}\right)\leq C\left(m_{1}^{\prime },\cdots ,m_{n_{2}}^{\prime }\right),$$
  where the equality only holds in case 1.
3. $C\left(m_{1},\underset{n}{\underset{︸}{\infty \cdots ,\infty }}\right)=C\left(\underset{n+1}{\underset{︸}{m_{1}+1,\cdots ,m_{1}+1}}\right)$.
4. For two *n*-TUB systems $S(m_{1},\cdots ,m_{n})$ and $S(m_{1}^{\prime },\cdots ,m_{n}^{\prime })$, if $m_{j}=m_{j}^{\prime }$ for 1≤j≤k and $m_{k+1}<m_{k+1}^{\prime }$, then
  $$C\left(m_{1},\cdots ,m_{n}\right)\leq C\left(m_{1}^{\prime },\cdots ,m_{n}^{\prime }\right),$$
  where the equality only holds in case 3.

This Main Theorem extends the results in [15] from DUB systems to general *n*-TUB systems. As *n* grows, the algebraic derivations and analysis on the related equations required for the proof become substantially more complex than in the case of n=2. In addition, it is not easy to find the equality conditions for *n*-TUB systems from the identity C(p,∞)=C(p+1,p+1) in DUB systems. For example, the relationship between $C(m_{1},m_{2},\underset{n-1}{\underset{︸}{\infty ,\cdots ,\infty }})$ and $C(m_{1},\underset{n}{\underset{︸}{m_{2}+1\cdots ,m_{1}+1}})$ is far from obvious.

In the next section, we will give the proof of the Main Theorem which is divided into several propositions. More precisely, Item 1 is proved by Proposition 3; Item 2 is proved by Propositions 3 and 4; Item 3 is proved by Proposition 5; and Item 4 is proved by Propositions 5 and 6.

## 2. Proof of the Main Results

To prove the Main Theorem, let us do some preliminary work first.

For a bilateral or unilateral *n*-TUB system $S(m_{1},\cdots ,m_{n})$, let Λ be the set of all *M*-length codes in $S(m_{1},\cdots ,m_{n})$, where $M=\max\{m_{k};m_{k}\;\mathrm{is}\;\mathrm{finite}\}$. Consider Λ as a finite symbol set, denoted by $\Lambda ={\left\{\beta_{j}\right\}}_{j\in J}$. We can define the transition matrix $B={\left(B_{ij}\right)}_{i,j\in J}$ as follows. For any two codes $\beta_{i}=z_{1}z_{2}\cdots z_{M}$ and $\beta_{j}=w_{1}w_{2}\cdots w_{M}$ in Λ, define $B_{ij}≜B\left(\beta_{i},\beta_{j}\right)=1$ if $z_{2}\cdots z_{M}=w_{1}\cdots w_{M-1}$ and $z_{1}z_{2}\cdots z_{M}w_{M}$ is an (M+1)-length code in $S(m_{1},\cdots ,m_{n})$; otherwise, define $B_{ij}≜B\left(\beta_{i},\beta_{j}\right)=0$. The transition matrix *B* completely describes whether $\beta_{i}$ and $\beta_{j}$ could be adjacent. Then, we obtain a subshift of finite type $\left(\Sigma_{B},\sigma \right)$ with transition matrix *B*, where

$$\Sigma_{B}=\left\{\left(x_{i}\right)\in \Lambda^{Z}\left(\;\mathrm{or}\;\Lambda^{N}\right);\;B\left(x_{i},x_{i+1}\right)=1\;\mathrm{for}\;\mathrm{all}\;i\in Z\;\left(\;\mathrm{or}\;N\right)\right\}$$

and σ is the shift on $\Sigma_{B}$. As is well known in symbolic dynamic systems, $\left(\Sigma_{B},\sigma \right)$ is topologically conjugate to the *n*-TUB systems $\left(S\left(m_{1},\cdots ,m_{n}\right),\sigma \right)$. Furthermore, if λ is the spectral radius of *B*, then

$$C(m_{1},\cdots ,m_{n})=\ln\lambda .$$

**Remark 1.** 
*Let n>2; define the map ϕ:{1,⋯,n}→{1,2} by ϕ(1)=1 and ϕ(k)=2 for any k=2,⋯,n. Then ϕ induces a map $h:S\left(m_{1},\cdots ,m_{n}\right)\to S\left(m_{1},\infty \right)$ which puts each symbol k in {1,⋯,n} to ϕ(k). One can see h is a continuous surjective and h∘σ=σ∘h. Therefore, h is a topological semi-conjugate from $\left(S\left(m_{1},\cdots ,m_{n}\right),\sigma \right)$ to $\left(S\left(m_{1},\infty \right),\sigma \right)$. By [15], $C\left(m_{1},\infty \right)\leq C\left(m_{1},\cdots ,m_{n}\right)$.*

By Remark 1, we have $C(m_{1},\cdots ,m_{n})>0$ except for C(1,1)=0.

To determinate the topological entropy of *n*-TUB systems, let us review some conclusions in Perron–Frobenius theory. We begin with the definition of a primitive matrix.

**Definition 1** 
(see [6]). *Let A be a non-negative matrix. For any index i, the period of state i, denoted by per(i), is defined as the greatest common divisor of those integers n≥1 for which ${\left(A^{n}\right)}_{ii}>0$, i.e.,*

$$\mathrm{per}\left(i\right):=\gcd\left\{n\geq 1;\;{\left(A^{n}\right)}_{ii}>0\right\}.$$

*If per(i)=1 for every index i, the matrix A is called an aperiodic matrix. If, for each ordered pair of indices (i,j), there exists some integer n≥1 such that ${\left(A^{n}\right)}_{ij}>0$, the matrix A is called an irreducible matrix. Furthermore, the matrix A is said to be primitive if A is irreducible and aperiodic.*

The following characterization of primitive matrices is useful.

**Lemma 1** 
([16]). *Let B≥0 be a square matrix. Then $B^{N}>0$ for some positive integer N if and only if B is primitive.*

Let $A_{p}$ be the number of *p*-length codes in $S(m_{1},\cdots ,m_{n})$; denote by $a_{p_{k}}$ the number of *p*-length codes ending with *k* in $S(m_{1},\cdots ,m_{n})$; then $A_{p}=\sum_{k=1}^{n}a_{p_{k}}$.

**Lemma 2** 
([17]). *Suppose that B is a primitive non-negative square matrix. Let λ be the spectral radius of B. Then*

$$\begin{array}{c} \lim_{p\to \infty }\frac{B^{p}}{\lambda^{p}}=rl, \end{array}$$

*where r and l are the right and left eigenvectors for B normalized so that rl=1.*

**Proposition 1.** 
*The transition matrix B defined as above is primitive. Furthermore, the limit $\lim_{p\to \infty }\frac{a_{p_{k}}}{A_{p}}$ exists for 1≤k≤n.*

**Proof.**  Since the square matrix *B* is a (0,1)-matrix, let N=M+2; we will prove that $B^{N}>0$. For any two *M*-length codes $\beta_{i}=\left(z_{1},z_{2},\cdots ,z_{M}\right)$ and $\beta_{j}=\left(w_{1},w_{2},\cdots ,w_{M}\right)$, if $z_{M}=y$ and $w_{1}=l$ where 1≤y, l≤n, let 1≤m≤n where *m* is equal to neither *y* nor *l*; then

$$C=(z_{1},z_{2},\cdots ,y,m,l,w_{2},\cdots ,w_{M})$$

is an (M+2)-length code from $\beta_{i}$ to $\beta_{j}$ in $\Sigma_{B}$ and hence ${\left(B^{N}\right)}_{ij}>0$. Notice that $A_{p+M}$ is the sum of all elements in $B^{p}$ and $a_{{\left(p+M\right)}_{k}}$ represents the sum of elements in some certain columns of $B^{p}$; then, by Lemma 2, the limits $\lim_{p\to \infty }\frac{a_{p_{k}}}{A_{p}}$ exist for 1≤k≤n.    □

Denote $\lim_{p\to \infty }\frac{a_{p_{k}}}{A_{p}}=x_{k}$; then $\sum_{k=1}^{n}x_{k}=1$. In addition, if λ is the spectral radius of *B*, then

$$\lim_{p\to \infty }\frac{A_{p+1}}{A_{p}}=\lambda .$$

For $0<m_{1}\leq \cdots \leq m_{n}<\infty$, we have, for each p∈N,

$$a_{{\left(p+1\right)}_{k}}=\sum_{j=1,j\neq k}^{n}\left(a_{p_{j}}+\cdots +a_{{\left(p-m_{k}+1\right)}_{j}}\right).$$

As p→∞,

$$\lambda^{m_{k}}x_{k}=\sum_{j=1,j\neq k}^{n}\sum_{q=1}^{m_{k}}\lambda^{m_{k}-q}x_{j}.$$

By Remark 1, it is easy to see λ>1 except for C(1,1); here we just consider the cases except for C(1,1) since we know C(1,1)=0. Therefore,

$$x_{k}=\frac{\lambda^{m_{k}-1}+\lambda^{m_{k}-2}+\cdots +\lambda +1}{\lambda^{m_{k}}+\lambda^{m_{k}-1}+\lambda^{m_{k}-2}+\cdots +\lambda +1}=\frac{\lambda^{m_{k}}-1}{\lambda^{m_{k}+1}-1}.$$

Since $\sum_{k=1}^{n}x_{k}=1$, we have

(1) $$\frac{\lambda^{m_{1}}-1}{\lambda^{m_{1}+1}-1}+\cdots +\frac{\lambda^{m_{n}}-1}{\lambda^{m_{n}+1}-1}=1$$

If $0<m_{1}\leq m_{2}\cdots \leq m_{n-1}<m_{n}=\infty$, similarly we have

$$a_{{\left(p+1\right)}_{k}}=\sum_{j=1,j\neq k}^{n-1}\left(a_{p_{j}}+\cdots +a_{{\left(p-m_{k}+1\right)}_{j}}\right)$$

and

$$a_{{\left(p+1\right)}_{n}}=A_{p}.$$

As p→∞,

$$\lambda^{m_{k}}x_{k}=\sum_{j=1,j\neq k}^{n-1}\sum_{q=1}^{m_{k}}\lambda^{m_{k}-q}x_{j}$$

and $\lambda x_{n}=1$. Therefore,

$$\frac{\lambda^{m_{1}}-1}{\lambda^{m_{1}+1}-1}+\cdots +\frac{\lambda^{m_{n-1}}-1}{\lambda^{m_{n-1}+1}-1}+\frac{1}{\lambda }=1$$

In general, for $0<m_{1}\leq \cdots <m_{t}=\cdots =m_{n}=\infty$, where 1<t≤n−1, λ satisfies the following equation:

(2) $$\frac{\lambda^{m_{1}}-1}{\lambda^{m_{1}+1}-1}+\cdots +\frac{\lambda^{m_{t-1}}-1}{\lambda^{m_{t-1}+1}-1}+\frac{n-t+1}{\lambda }=1.$$

**Proposition 2.** 
*For $\left(m_{1},\cdots ,m_{n}\right)\neq \left(1,\cdots ,1\right),\left(\infty ,\cdots ,\infty \right)$, there exists one and only one root λ of the characteristic Equation (1) or (2) in the open interval (n−1,n) and $C(m_{1},\cdots ,m_{n})=\ln\lambda$.*

**Proof.**  For k=1,⋯,n,

$$f_{k}\left(\lambda \right)=\frac{\lambda^{m_{k}}-1}{\lambda^{m_{k}+1}-1}$$

is a strictly decreasing function since $f_{k}^{\prime }\left(\lambda \right)<0$ for λ>1. Then

$$F\left(\lambda \right)=\frac{\lambda^{m_{1}}-1}{\lambda^{m_{1}+1}-1}+\cdots +\frac{\lambda^{m_{n}}-1}{\lambda^{m_{n}+1}-1}$$

is a strictly decreasing function in (n−1,n).

For a fixed $\lambda_{0}$, let

$$\begin{array}{c} g_{m_{k}}\left(\lambda_{0}\right)=\frac{{\lambda_{0}}^{m_{k}}-1}{{\lambda_{0}}^{m_{k}+1}-1},\;g_{\infty }\left(\lambda_{0}\right)=\frac{1}{\lambda_{0}}, \end{array}$$

we have $g_{m_{k}+1}\left(\lambda_{0}\right)-g_{m_{k}}\left(\lambda_{0}\right)>0$ and $g_{\infty }\left(\lambda_{0}\right)-g_{m_{k}}\left(\lambda_{0}\right)>0$ (see [15], page 310). Then

$$\begin{array}{c} F\left(\lambda \right)>n\cdot g_{1}\left(\lambda \right)=\frac{n(\lambda -1)}{\lambda^{2}-1}=\frac{n}{\lambda +1}\;\mathrm{and}\;F\left(n-1\right)>1. \end{array}$$

Notice that $\frac{n^{m_{k}}-1}{n^{m_{k}+1}-1}<\frac{1}{n}$ holds for every 1≤k≤n; when n≥2, we have

$$F\left(n\right)=\frac{n^{m_{1}}-1}{n^{m_{1}+1}-1}+\cdots +\frac{n^{m_{n}}-1}{n^{m_{n}+1}-1}<n\cdot \frac{1}{n}=1.$$

Therefore, the characteristic Equation (1) has a unique root in the open interval (n−1,n). Similarly, the characteristic Equation (2) has a unique root in the open interval (n−1,n) since $\frac{\lambda^{m_{i}}-1}{\lambda^{m_{i}+1}-1}<\frac{1}{\lambda }$ for 1≤i<t. It follows from the discussions before this theorem that the unique root is the spectral radius of *B* corresponding to $S(m_{1},\cdots ,m_{n})$ and hence

$$C(m_{1},\cdots ,m_{n})=\ln\lambda .\;\;\;\;$$

   □

Now, let us consider the assertions of the Main Theorem.

**Proposition 3.** 
*For n-TUB system S(∞,∞⋯,∞) and (n+1)-TUB system S(1,1⋯,1,1) we have*

C(∞,∞⋯,∞)=C(1,1⋯,1,1)=lnn.

**Proof.**  For the *n*-TUB system S(∞,∞⋯,∞), its characteristic equation is

(3) $$\frac{n}{\lambda }=1,$$

then λ=n.

For the (n+1)-TUB system S(1,1⋯,1,1), its characteristic equation is

(4) $$\frac{\left(n+1)(\lambda -1\right)}{\lambda^{2}-1}=1,$$

then the spectral radius is λ=n. Therefore,

C(∞,∞⋯,∞)=C(1,1⋯,1,1)=lnn.

   □

**Proposition 4.** 
*For any $m_{1}\leq m_{2}\leq \cdots \leq m_{n}<m_{n}^{\prime }\leq \infty$, we have*

$$C\left(m_{1},\cdots ,m_{n-1},m_{n}\right)<C\left(m_{1},\cdots ,m_{n-1},m_{n}^{\prime }\right).$$

**Proof.**  The proof is similar to Proposition 2.6 in [15]. Let $\lambda_{0},\lambda_{1}\in \left(n-1,n\right)$ with $C\left(m_{1},\cdots ,m_{n-1},m_{n}\right)=\ln\lambda_{0}$ and $C\left(m_{1},\cdots ,m_{n-1},m_{n}^{\prime }\right)=\ln\lambda_{1}$. Let $g_{m_{n}}\left(\lambda \right)=\frac{\lambda^{m_{n}}-1}{\lambda^{m_{n}+1}-1}$ for finite positive integer $m_{n}$ and $g_{\infty }\left(\lambda \right)=\frac{1}{\lambda }$. For $\lambda_{0}\in \left(n-1,n\right)$, one can see

$$\begin{array}{cc} g_{m_{n}+1}\left(\lambda_{0}\right)-g_{m_{n}}\left(\lambda_{0}\right)= & \frac{\lambda_{0}^{m_{n}+1}-1}{\lambda_{0}^{m_{n}+2}-1}-\frac{\lambda_{0}^{m_{n}}-1}{\lambda_{0}^{m_{n}+1}-1}\\ = & \frac{\lambda_{0}^{m_{n}}{\left(\lambda_{0}-1\right)}^{2}}{\left(\lambda_{0}^{m_{n}+2}-1\right)\left(\lambda_{0}^{m_{n}+1}-1\right)}\\ > & 0, \end{array}$$

and

$$g_{\infty }\left(\lambda_{0}\right)-g_{m_{n}}\left(\lambda_{0}\right)=\frac{\lambda_{0}-1}{\lambda_{0}\left(\lambda_{0}^{m_{n}+1}-1\right)}>0.$$

Notice that $\lambda_{0}$ belongs to (n−1,n) and satisfies Equation (1), i.e.,

$$\frac{\lambda_{0}^{m_{1}}-1}{\lambda_{0}^{m_{1}+1}-1}+\cdots +\frac{\lambda_{0}^{m_{n}}-1}{\lambda_{0}^{m_{n}+1}-1}=1.$$

Then, if $m_{n}<m_{n}^{\prime }<\infty$,

$$\frac{\lambda_{0}^{m_{1}}-1}{\lambda_{0}^{m_{1}+1}-1}+\cdots +\frac{\lambda_{0}^{m_{n}^{\prime }}-1}{\lambda_{0}^{m_{n}^{\prime }+1}-1}>1\;\;\mathrm{and}\;\;\frac{\lambda_{1}^{m_{1}}-1}{\lambda_{1}^{m_{1}+1}-1}+\cdots +\frac{\lambda_{1}^{m_{n}^{\prime }}-1}{\lambda_{1}^{m_{n}^{\prime }+1}-1}=1,$$

and, if $m_{n}^{\prime }=\infty$,

$$\frac{\lambda_{0}^{m_{1}}-1}{\lambda_{0}^{m_{1}+1}-1}+\cdots +\frac{\lambda_{0}^{m_{n-1}}-1}{\lambda_{0}^{m_{n-1}+1}-1}+\frac{1}{\lambda_{0}}>1\;\;\mathrm{and}\;\;\frac{\lambda_{1}^{m_{1}}-1}{\lambda_{1}^{m_{1}+1}-1}+\cdots +\frac{\lambda_{1}^{m_{n-1}}-1}{\lambda_{1}^{m_{n-1}+1}-1}+\frac{1}{\lambda_{1}}=1.$$

Since the functions $\frac{\lambda^{m_{1}}-1}{\lambda^{m_{1}+1}-1}+\cdots +\frac{\lambda^{m_{n}}-1}{\lambda^{m_{n}+1}-1}$ and $\frac{\lambda^{m_{1}}-1}{\lambda^{m_{1}+1}-1}+\cdots +\frac{\lambda^{m_{n-1}}-1}{\lambda^{m_{n-1}+1}-1}+\frac{1}{\lambda }$ are strictly decreasing on (n−1,n), we have $\lambda_{0}<\lambda_{1}$. In conclusion,

$$C\left(m_{1},\cdots ,m_{n-1},m_{n}\right)<C\left(m_{1},\cdots ,m_{n-1},m_{n}^{\prime }\right).$$

   □

**Proposition 5.** 
*For $1\leq m_{1}\leq \infty$, $C\left(m_{1},\underset{n}{\underset{︸}{\infty \cdots ,\infty }}\right)=C\left(\underset{n+1}{\underset{︸}{m_{1}+1,\cdots ,m_{1}+1}}\right)$.*

**Proof.**  Denote $\ln\lambda_{1}=C\left(m_{1},\underset{n}{\underset{︸}{\infty \cdots ,\infty }}\right)$ and $\ln\lambda_{2}=C\left(\underset{n+1}{\underset{︸}{m_{1}+1,\cdots ,m_{1}+1}}\right)$. Then, $\lambda_{1}$ satisfies the following equation:

$$\frac{\lambda^{m_{1}}-1}{\lambda^{m_{1}+1}-1}+\frac{n-1}{\lambda }=1,$$

which is equivalent to

$$\frac{\lambda^{m_{1}+2}-n\lambda^{m_{1}+1}+n-1}{\lambda (\lambda^{m_{1}+1}-1)}=0.$$

Similarly, $\lambda_{2}$ satisfies the following equation:

$$\frac{\lambda^{m_{1}+1}-1}{\lambda^{m_{1}+2}-1}=\frac{1}{n},$$

which is equivalent to

$$\frac{\lambda^{m_{1}+2}-n\lambda^{m_{1}+1}+n-1}{n(\lambda^{m_{1}+2}-1)}=0.$$

Then, both $\lambda_{1}$ and $\lambda_{2}$ satisfy the equation $\lambda^{m_{1}+2}-n\lambda^{m_{1}+1}+n-1=0$. Notice that $\lambda_{1},\lambda_{2}\in \left(n,n+1\right)$. It follows from Proposition 2 that $\lambda_{1}=\lambda_{2}$ and hence

$$C\left(m_{1},\underset{n}{\underset{︸}{\infty \cdots ,\infty }}\right)=C\left(\underset{n+1}{\underset{︸}{m_{1}+1,\cdots ,m_{1}+1}}\right).$$

   □

**Proposition 6.** 
*For any integer n>2 and 2≤s≤n satisfying*

$$m_{1}\leq m_{2}\leq \cdots \leq m_{s}<\infty ,$$

*we have*

$$\begin{array}{c} C\left(m_{1},\cdots ,m_{s-1},m_{s},\underset{n-s}{\underset{︸}{\infty \cdots ,\infty }}\right)<C\left(m_{1},\cdots ,m_{s-1},\underset{n-s+1}{\underset{︸}{m_{s}+1,\cdots ,m_{s}+1}}\right). \end{array}$$

**Proof.**  For $S(m_{1},\cdots ,m_{s-1},m_{s},\infty ,\cdots \infty )$, we have

$$M\left(\lambda \right)=\frac{\lambda^{m_{1}}-1}{\lambda^{m_{1}+1}-1}+\cdots +\frac{\lambda^{m_{s}}-1}{\lambda^{m_{s}+1}-1}+\frac{n-s}{\lambda }=1,$$

which is equivalent to  

$$\frac{\sum_{k=1}^{s-1}\prod_{j\neq k}^{s-1}\left(\lambda^{m_{j}+1}-1\right)\left(\lambda^{m_{k}}-1\right)}{\prod_{i=1}^{s-1}\left(\lambda^{m_{i}+1}-1\right)}+\frac{\lambda^{m_{s}}-1}{\lambda^{m_{s}+1}-1}+\frac{n-s}{\lambda }=1,$$

and, furthermore, is equivalent to

(5) $$\begin{array}{cc} \; & \left(\sum_{k=1}^{s-1}\prod_{j\neq k}^{s-1}\left(\lambda^{m_{j}+1}-1\right)\left(\lambda^{m_{k}}-1\right)\right)\left(\lambda^{m_{s}+1}-1\right)\lambda +\left(\prod_{i=1}^{s-1}\left(\lambda^{m_{i}+1}-1\right)\right)\left(\lambda^{m_{s}}-1\right)\lambda \\ + & \left(n-s\right)\prod_{i=1}^{s}\left(\lambda^{m_{i}+1}-1\right)-\lambda \prod_{i=1}^{s}\left(\lambda^{m_{i}+1}-1\right)=0. \end{array}$$

For $S(m_{1},\cdots ,m_{s-1},m_{s}+1,\cdots ,m_{s}+1)$, we have

$$\begin{array}{c} N\left(\lambda \right)=\frac{\lambda^{m_{1}}-1}{\lambda^{m_{1}+1}-1}+\cdots +\frac{\lambda^{m_{s-1}}-1}{\lambda^{m_{s-1}+1}-1}+\frac{\left(n-s+1\right)\left(\lambda^{m_{s}+1}-1\right)}{\lambda^{m_{s}+2}-1}=1, \end{array}$$

which is equivalent to

$$\frac{\sum_{k=1}^{s-1}\prod_{j\neq k}^{s-1}\left(\lambda^{m_{j}+1}-1\right)\left(\lambda^{m_{k}}-1\right)}{\prod_{i=1}^{s-1}\left(\lambda^{m_{i}+1}-1\right)}+\frac{\left(\lambda^{m_{s}+1}-1\right)\left(n-s+1\right)}{\lambda^{m_{s}+2}-1}=1$$

and, furthermore, is equivalent to

(6) $$\begin{array}{cc} \; & \left(\sum_{k=1}^{s-1}\prod_{j\neq k}^{s-1}\left(\lambda^{m_{j}+1}-1\right)\left(\lambda^{m_{k}}-1\right)\right)\left(\lambda^{m_{s}+2}-1\right)+\left(\prod_{i=1}^{s-1}\left(\lambda^{m_{i}+1}-1\right)\right)\cdot \left(\lambda^{m_{s}+1}-1\right)\left(n-s+1\right)\\ - & \left(\prod_{i=1}^{s-1}\left(\lambda^{m_{i}+1}-1\right)\right)\left(\lambda^{m_{s}+2}-1\right)=0. \end{array}$$

Let f(λ) and g(λ) denote the functions of the left side in Equations (5) and (6), respectively. Then,

$$h\left(\lambda \right)=f\left(\lambda \right)-g\left(\lambda \right)=\left(\sum_{k=1}^{s-1}\prod_{j\neq k}^{s-1}\left(\lambda^{m_{j}+1}-1\right)\left(\lambda^{m_{k}}-1\right)\right)\left(\lambda -1\right).$$

Assume that $\lambda_{0}$ is the only solution of Equation (5) in the interval (n−1,n), that is, $f(\lambda_{0})=0$. Then

$$h\left(\lambda_{0}\right)=-g\left(\lambda_{0}\right)=\left(\sum_{k=1}^{s-1}\prod_{j\neq k}^{s-1}\left(\lambda_{0}^{m_{j}+1}-1\right)\left(\lambda_{0}^{m_{k}}-1\right)\right)\left(\lambda_{0}-1\right)>0.$$

Consequently, we have $g(\lambda_{0})<0$; this implies $N(\lambda_{0})>1$. Therefore, the only root $\lambda_{1}$ of (5) is bigger than $\lambda_{0}$.    □

**Proof**  (Proof of the Main Theorem)**.** By Propositions 2, 3, 4, 5 and 6, we complete the proof of the Main Theorem. Actually, we can order all the *n*-TUB systems by their topology entropy as follows:  

$$\begin{array}{cc} \ln(n-1)= & C(1,\cdots ,1,1)<C(1,\cdots ,1,2)<\cdots \\ < & C(1,\cdots ,1,\infty )<C(1,\cdots ,2,2)<\cdots \\ < & C(1,\cdots ,1,\infty ,\infty )<C(1,\cdots ,1,2,2,2)<\cdots \\ < & C(1,\infty ,\cdots ,\infty )=C(2,2,\cdots ,2,2)<\cdots \\ < & C(\infty ,\cdots ,\infty )=\ln n. \end{array}$$

   □

## 3. Experimental Validation

We present an algorithm to compute the transition matrices and eigenvalues of the *n*-TUB systems. Numerical examples are then provided to validate the derived theorems. Our code is available at https://github.com/HeJiaxing-hjx/N_tuple_UB.git (accessed on 30 December 2025).

To compute the topological entropy, we employ the power iteration method to find the spectral radius ρ(B) of the transition matrix. Since *B* is non-negative, the Perron–Frobenius theorem guarantees that the largest eigenvalue is real and positive, representing the exponential growth rate of the number of admissible words.

Let *n* denote the cardinality of the alphabet and *L* be the target sequence length. The total number of generated admissible sequences is denoted by $N_{B}=\left|P\right|$. During the sequence generation stage, the backtracking tree contains $O(n^{L})$ nodes, with each node requiring O(L) time, thus leading to a time complexity of $O(n^{L}\cdot L)$ for this step. In the primitive matrix construction stage, initializing the primitive matrix *B* takes $O(N_{B}^{2})$ time, while the string matching and constraint verification within the loop require O(L·n) time. Consequently, the time complexity of this stage is $O(N_{B}^{2}\cdot L\cdot n)$. Let *I* denote the number of iterations required by the power iteration method. When using the power iteration method to calculate the maximum eigenvalue λ of the $N_{B}\times N_{B}$ matrix *B*, each dense matrix–vector multiplication requires $O(N_{B}^{2})$ time. Therefore, the total time required is $O(I\cdot N_{B}^{2})$. The overall time complexity is $O(n^{L}\cdot L+N_{B}^{2}\cdot L\cdot n+I\cdot N_{B}^{2})$.

By applying Algorithms 1 and 2, the topological entropy for the following example is computed as follows.

**Example 1.** 
$$\begin{array}{cc} \; & C(\infty ,\infty ,\infty )=\;C(1,1,1,1)\approx 1.0986,\\ & C(1,3,3,4)\approx 1.3240,\;\;C(2,2,2,3)\approx 1.3437,\\ & C(2,2,2,\infty )\approx 1.3474,\;C(2,2,3,3)\approx 1.3542,\\ \; & C(2,\infty ,\infty ,\infty )=\;C(3,3,3,3)\approx 1.3741. \end{array}$$

*Then we have*

$$\begin{array}{cc} \; & C(\infty ,\infty ,\infty )=C(1,1,1,1)<C(1,3,3,4)\\ < & C(2,2,2,3)<C(2,2,2,\infty )<C(2,2,3,3)\\ < & C(2,\infty ,\infty ,\infty )=C(3,3,3,3), \end{array}$$

*which validates our Main Theorem.*

In Example 1, the computation time for C(∞,∞,∞) with a length of 3 is 9 ms, whereas that for C(∞,∞,∞,∞) with a length of 4 is approximately 365 ms. It can be observed that, when the length of code equals the alphabet cardinality, the computation time increases significantly as the alphabet cardinality grows. Furthermore, the computation times for C(∞,∞,∞) are 13 ms at a length of 4 and 409 ms at a length of 5. This indicates that, given the same alphabet cardinality, a longer length of code leads to a longer computation time.
**Algorithm 1:** Generate Admissible Sequences
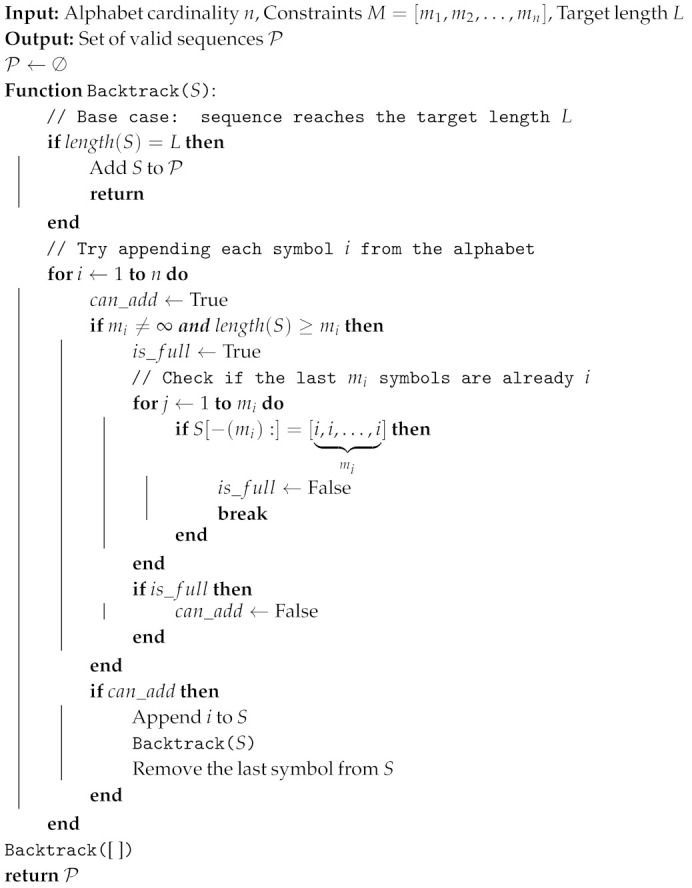


**Algorithm 2:** Construction of the Transition Matrix *B* and Calculating Its Topological Entropy

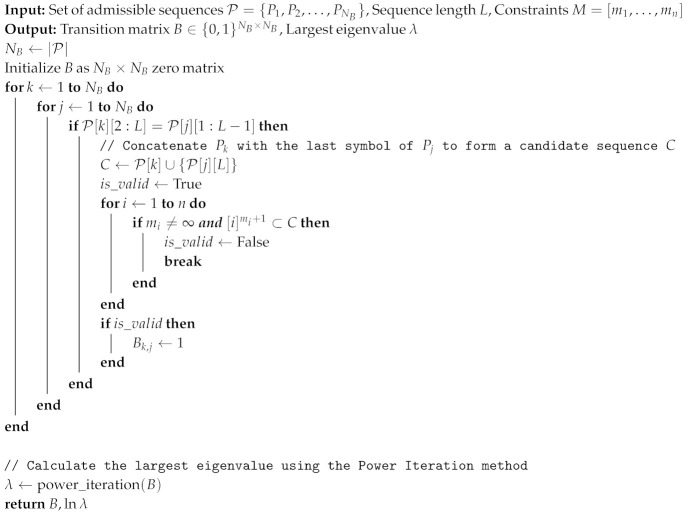



## Data Availability

Our code is available at https://github.com/HeJiaxing-hjx/N_tuple_UB.git (accessed on 30 December 2025).
